# Infrared Spectroscopy of Protonated Water Clusters via the Quantum Thermal Bath Method and Highly Accurate Machine-Learned Potentials

**DOI:** 10.1021/acs.jpca.6c01687

**Published:** 2026-07-22

**Authors:** T. Baird, R. Vuilleumier, S. Bonella

**Affiliations:** † Centre Européen de Calcul Atomique et Moléculaire (CECAM), Ecole Polytechnique Fédérale de Lausanne, 1015 Lausanne, Switzerland; ‡ École Normale Supérieure, PSL University, Sorbonne Université, CNRS, UMR 8228 Chimie Physique et Chimie du Vivant (CPCV), 75005 Paris, France

## Abstract

The spectral features of water clusters provide important information on their structure and dynamics and can assist in deciphering the nature of the local environment of aqueous solutions in a variety of different conditions. Accurately capturing these features via numerical simulations is a nontrivial task that typically requires a sophisticated combination of high-level electronic structure methods and costly quantum dynamics techniques. We present results of molecular dynamics simulations of the IR spectra of protonated water clusters, ranging from the monomer to the tetramer, obtained via the combination of highly accurate machine-learned potential energy surfaces (PES) and dipole moment surfaces (DMS), and the quantum thermal bath (QTB) methodology which facilitates cost-effective inclusion of NQEs in molecular dynamics simulations. We compare our results with previous theoretical and experimental studies and show that this combination provides a significantly cheaper, yet still suitably accurate, alternative to approaches such as ring polymer molecular dynamics and its thermostated variant (TRPMD), often used for these calculations.

## Graphical abstract

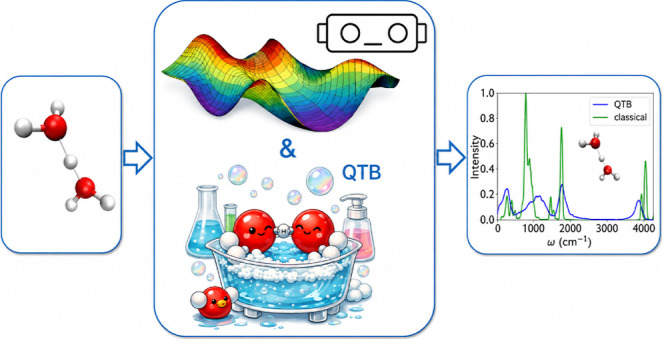

## 1 Introduction

The investigation of water clusters holds significant theoretical and practical importance due to the ubiquitous presence of water in nature and its crucial role in many chemical and biological processes. The spectral properties of water clusters offer insights not only into the structure and dynamics of these clusters but carry also signatures that help in understanding the behavior of water across different environments, including at interfaces or under confinement.^1,2^ Furthermore, systematic studies of small water clusters also provide information about proton transfer, a process of key importance in chemistry and physics. Finally, the infrared (IR) spectra of water clusters are highly sensitive to their internal hydrogen bonding network, making IR spectroscopy a valuable tool for probing intermolecular interactions among the molecules which constitute these clusters.^3,4^

Accurately modeling these intermolecular interactions is essential for capturing the various molecular motions that give rise to spectral features. As a result, in computational studies, high-precision potential energy surfaces and force fields are required for reliable spectral calculations. Similarly, the accuracy of dipole moment estimates is critical for faithfully reproducing the spectral peak positions and relative intensities. This necessity, in turn, demands sophisticated and computationally expensive electronic structure methods to accurately determine the system’s charge density and dipole moments. The high computational cost associated with *first-principles* calculations has led to considerable efforts to develop analytical models for the potential and dipole moment surfaces for different systems.^5–10^ More recently, machine learning techniques have emerged as a powerful alternative to design many body potentials from accurate electronic structure calculations, in particular for water from clusters to bulk.^11–20^

Incorporating nuclear quantum effects (NQEs) is also essential for accurately reproducing key infrared spectral features of water clusters.^21^ These include the characteristic red shift of peaks relative to classical predictions and the presence of combination bands. Different numerical strategies have been adopted to account for NQEs. Notably, spectra have been computed using both the multiconfigurational time-dependent Hartree (MCTDH) and vibrational self-consistent field/vibrational configuration interaction (VSCF/VCI) methods to solve the nuclear Schrödinger equation.^8,22–25^

These approaches, in their standard form, do not account for thermal effects, but they can be extended so as to incorporate the influence of temperature. VSCF/VCI achieves this in an *a posteriori* fashion by weighting computed transitions from thermally populated excited states according to the relevant Boltzmann weights.^26^ The extension of VSCF/VCI to “hot bands”, however, does not by itself include thermally induced structural sampling, dynamical dephasing, or temperature-dependent exploration of the potential-energy surface.^27^ This should be distinguished from finite-temperature MD spectra, wherein temperature enters through thermally sampled trajectories and time-correlation functions; MCTDH, on the other hand, can be modified to go beyond its original pure-state wavepacket formulation to instead work with thermal density operators, purified thermal wave functions, or an ensemble of thermal wavepackets.^28–30^ In practice, however, the high-level VSCF/VCI and MCTDH benchmarks most often used for the protonated water clusters considered here are effectively zero-temperature or state-resolved calculations, e.g. spectra obtained from the vibrational ground state or from low-lying eigenstates.^8,23,31,32^ Extending these calculations to finite temperature would require converging additional thermally populated initial states and their corresponding sets of transitions; for VSCF/VCI this rapidly enlarges the state space. Meanwhile, finite-temperature MCTDH variants introduce computational overheads associated with purification strategies, the inherent increase in complexity due to use of density matrices, or stochastic sampling requirements. These introduce costs that grow with temperature and system size.^26,30,32^ A conceptually simpler route to finite-temperature spectra is classical molecular dynamics. This approach naturally includes thermal sampling through coupling with a thermostat, but it also exaggerates temperature effects in high-frequency vibrations because equipartition assigns thermal energy to every classical degree of freedom, whereas in a quantum system only modes with excitation energies comparable to *k*_B_ *T* are substantially thermally populated at low temperature. Alternative finite-temperature approaches based either on semiclassical methods^33,34^ or path integral molecular dynamics (PIMD)^35,36^ have therefore been proposed. Standard imaginary-time PIMD samples quantum equilibrium distributions but does not by itself provide real-time vibrational spectra; practical spectral calculations instead use dynamical extensions such as RPMD, TRPMD, CMD, or QCMD.^35–37^ While these approaches do provide interesting insights, they are known to face limitations due to the combination of classical dynamics and quantum probability distribution sampling (semiclassical) or may become quite expensive in deep quantum regimes and fail to reproduce, for example, the correct trends for peaks’ intensities at low temperature without bespoke corrections.^34,38^

In this paper, we revisit the spectroscopy of protonated water clusters at finite temperature combining a state-of-the-art ML representation of the interactions and the dipole moment surface recently developed in the group of D. Marx^39,40^ with the quantum thermal bath (QTB)^41^ approach. We compare the resulting spectra with those obtained from classical MD simulations and with previous numerical and experimental studies to evaluate the accuracy of this approach and assess the influence of NQEs on the spectral properties of water clusters. Our calculations illustrate strengths and weaknesses of QTB in comparison with experiments and MCTDH or VSCF/VCI calculations. We note that the MCTDH and VSCF/VCI reference calculations used below were themselves performed on high-level PESs and DMSs: the H_5_O_2_^+^ calculations of Vendrell et al. used the full-dimensional Huang–Braams–Bowman PES/DMS while the larger-cluster VSCF/VCI calculations used the Bowman-group many-body PES/DMSs developed for protonated water clusters.^9,21,23,32,42,43^ They also indicate that the approach produces spectra of comparable accuracy to (T)­RPMD at lower numerical cost. The main limitation of QTB and path integral based methods, namely broadening of peaks and consequent lack of resolution of finer features, is illustrated in our results and discussed in detail in the Section 4.

## 2 Methods

### 2.1 Quantum Thermal Bath Dynamics

QTB models quantum effects by coupling nuclear classical dynamics to a thermal reservoir endowed with a noise power spectral density that emulates quantum fluctuations.^41^ In practice, this involves solving numerically the generalized Langevin dynamics

1 $$m_{I}\frac{d^{2}R_{I}}{dt^{2}}=-\nabla_{R_{I}}E\left(R_{I}\right)-m_{I}\gamma \frac{dR_{I}}{dt}+F_{I}\left(t\right)$$

In the equation above, **R**_*I*_ are the Cartesian coordinates of ion *I*, and *m*_*I*_ is the mass of the ion. γ is the friction coefficient of the thermostat, and $F_{I}=\left[F_{I}^{x}F_{I}^{y}F_{I}^{z}\right]$ represents a three-dimensional random force acting on ion *I*, with the associated colored noise spectrum

2 $$C_{F_{J}^{\alpha }F_{K}^{\beta }}\left(\omega \right)=2m_{K}\gamma \theta \left(\omega ,T\right)\delta_{JK}\delta^{\alpha \beta },\alpha ,\beta \in x,y,z$$

where $C_{F_{J}^{\alpha }F_{K}^{\beta }}\left(\omega \right)$ denotes the autocorrelation function between the α-component of the force on ion *J* and the β-component on ion *K*. The function

3 $$\theta \left(\omega ,T\right)=\frac{\hbar \omega }{2\;\tanh\left(\frac{\hbar \omega }{2k_{B}T}\right)}$$

is the quantum mechanical power spectral density of a harmonic oscillator at temperature *T*. The QTB evolution, which is exact only for harmonic systems, can be justified based on an approximation to the path integral representation of the density matrix of a generic quantum system linearly coupled to a bath of quantum harmonic oscillators.^44^ Within the limits of this justification, QTB has proven accurate in a number of applications.^41,45–47^ Most notably, the approach performs well for the simulation of vibrational spectroscopy.^38^ A well-documented limitation of the Quantum Thermal Bath (QTB), also shared by other semiclassical approaches, is its susceptibility to zero-point energy leakage (ZPEL).^48^ This issue arises when the energy residing in high-frequency vibrational modes—initially distributed according to the quantum thermal statistics encoded by QTB—cascades into lower-frequency modes due to the classical time evolution that drives toward equipartition. An effective approach proposed to alleviate ZPEL is the adaptive quantum thermal bath (adQTB). adQTB monitors ZPEL on the fly via the diagnostic function proposed in^49^

4 $$\Delta_{FDT,i}\left(\omega \right)=Re\left[C_{vF,i}\left(\omega \right)\right]-m_{i}\gamma_{i}\left(\omega \right)C_{vv,i}\left(\omega \right)$$

where *C*_vF,*i*_(ω) and *C*_vv,*i*_(ω) denote the cross-correlation between velocity and random force, and the velocity autocorrelation, respectively, for the *i*-th Cartesian coordinate. Deviations from zero of the function above indicate violations of the quantum fluctuation dissipation theorem induced by ZPEL. These can then be mitigated by dynamically adjusting the friction coefficient γ in a frequency-dependent fashion, i.e., γ = γ­(ω), with the goal of minimizing the magnitude of Δ_FDT,*i*_(ω) across all modes and degrees of freedom.^49^

Note that, in contrast to (T)­RPMD methods, QTB does not require the introduction of replicas (or beads) to represent quantum effects. The high number of replicas needed to capture quantum effects, e.g., at low temperatures increases the cost of (T)­RPMD compared to classical calculations and is a key limiting factor for these approaches. The QTB numerical overhead is limited to the need to generate the colored noise in the generalized Langevin equation eq 1. Details on the QTB numerical implementation adopted in this work are presented in Appendix A. In Appendix D we report the timing of our QTB production runs, demonstrating that this overhead is negligible.

Notably, calculations presented in the next section did not show appreciable ZPEL, with the fluctuation–dissipation relation (eq 4) proving to be well-satisfied, and the IR spectra obtained using QTB and adQTB being in very good agreement for all systems considered. Consequently, in the following we report only the results obtained with standard QTB.

### 2.2 NN-PES/DMS and Computation of IR Spectra

To ensure a fast and accurate representation of the interactions and of the dipole moment surfaces for the water clusters, we adopted specifically designed machine-learned force fields and dipoles. We incorporated the Neural Network Potential (NNP) library^39,40^ (built on RubNNet4MD^50^) into the Tinker-HP molecular dynamics package.^51^ The Tinker-HP engine had a pre-existing extensive suite of functionality for both classical and quantum MD simulations, in particular providing support for QTB and adQTB, limiting our task to interfacing the NNP library.

The NNP library is based on the neural network architecture introduced by Behler and Parrinello^52^ and has been trained on a comprehensive data set of water cluster configurations (ranging from H_2_O to H_9_O_4_^+^) generated using ab initio molecular dynamics (AIMD) and path integral AIMD. The energies of these clusters were computed at a level of accuracy comparable to coupled-cluster CCSD­(T) theory. The NNP library yields accurate and transferable energies and forces for these systems, and it has been employed in multiple studies to successfully predict the static and dynamical properties of water clusters.^39^ Additionally, the library includes a dipole moment surface (DMS) trained on a data set of molecular configurations, with dipole moments computed at coupled-cluster accuracy. Use of the neural network drastically reduces the substantial computational cost associated with directly performing these high-level quantum chemistry calculations. The NNP-DMS approach thus enables an efficient yet accurate determination of charge distributions and dipole moments, which are essential for infrared (IR) spectral calculations.^31,40^

In all calculations reported in the following, the IR spectrum is obtained from the (Kubo-transformed) dipole derivative autocorrelation function. Specifically, during each simulation step, the total dipole moment of the system is predicted by passing the ionic geometry through the DMS. The dipole time derivative is then computed using finite differences and the Fourier transform of the dipole derivative autocorrelation function, $C_{\mathrm{\mu ̇}\mathrm{\mu ̇}}\left(\omega \right)$ , is subsequently determined. For QTB simulations, this autocorrelation function is Kubo-transformed using

5 $${\mathrm{C̃}}_{\mathrm{\mu ̇}\mathrm{\mu ̇}}\left(\omega \right)=\frac{\tanh\left(\beta \hbar \omega /2\right)}{\hbar \omega }C_{\mathrm{\mu ̇}\mathrm{\mu ̇}}\left(\omega \right)$$

and the IR spectrum is then computed using the Beer–Lambert law

6 $$n\left(\omega \right)\alpha \left(\omega \right)=\frac{\pi \beta }{3cV\varepsilon_{0}}{\mathrm{C̃}}_{\mathrm{\mu ̇}\mathrm{\mu ̇}}\left(\omega \right)$$

where *n*(ω) is the refractive index, α­(ω) is the absorption coefficient, *c* is the speed of light, *V* is the simulation box volume, and ε_0_ is the vacuum permittivity.

In this work, the final IR spectra are obtained by averaging the spectra derived from trajectory segments covering a frequency range of 0–15,000 cm^–1^ (spanning far-infrared to near-infrared regions, including overtone ranges). Each segment is smoothed using a Hann window function of width 750 cm^–1^ in order to mitigate ringing artifacts, and the initial trajectory segments (corresponding to approximately 25 ps of simulation time) are discarded to account for equilibration. Lastly, the final calculated spectrum is itself smoothed via a sliding window procedure, with the window size set to a value between 10 and 50 cm^–1^. This window size was chosen such that it is sufficiently small to avoid loss of resolution of any fine spectral features while simultaneously reducing the noisiness of the spectra.

Since theoretical IR spectra in the literature are often computed with the center-of-mass (COM) motion removed, we implemented a functionality to eliminate COM motion in our simulations when necessary. In our classical MD simulations, this was done conventionally by subtracting translational motion of the COM and global rotation contributions from atomic velocities at each time step. For QTB, however, the procedure is more delicate due to the need to preserve the quantum fluctuation–dissipation relation, which relies on all degrees of freedom. Details on our algorithm are provided in Appendix B.

## 3 Results

We have performed simulations of several systems of increasing size, namely: H_2_O, H_3_O^+^, H­(H_2_O)_2_^+^, H­(H_2_O)_3_^+^, H­(H_2_O)_4_^+^ (Eigen), H­(H_2_O)_4_^+^ (ring, *cis-*, and *trans*-Zundel). Computed IR spectra of a selection of these systems (H_2_O, H­(H_2_O)_2_^+^, H­(H_2_O)_3_^+^ and H­(H_2_O)_4_^+^ (Eigen)) are presented in the following sections, together with analysis of the main spectral features observed. The remaining clusters are discussed in Appendix C.

In all cases, simulations were performed in the gas phase and within the *NVT* ensemble, with different temperatures considered to allow comparison with existing experimental and theoretical results which we extracted via WebPlotDigitizer^53^ from the references indicated in each figure caption. The simulations were performed for a total of 1 ns (discarding the first 25 ps as equilibration), the time step was set to d*t* = 0.1 fs, and properties recorded every 10 fs. The small value of the time step improved the resolution of the spectral features in the higher frequency ranges considered. The ability to affordably obtain such long trajectory lengths facilitates significant improvements in spectral resolution and statistics. In the classical simulations, the temperature was controlled via Langevin dynamics, integrated using the BAOAB thermostat,^54^ with a friction coefficient of 2 ps^–1^. This choice of friction allowed for a good balance between enhancing ergodicity while minimizing the damping effect on the system’s dynamics. To further counteract the broadening effect which damping has on the spectrum, the deconvolution procedure first introduced by Rossi et al. was employed^55,56^ to all calculated spectra (both QTB and classical). In all plots, the spectra are normalized so that the highest peak of each individual spectrum included in the figure has a maximum value of 1. This choice is motivated by the need to present on somewhat equal footing comparisons between calculations for which results in absolute units are not always available. To facilitate comparisons with experimental data (or future calculations), we also provide the scaling factors needed to transform the intensities to absolute units in the figures’ captions. Intensities are scaled to the absorption coefficient *n*α corresponding to a reference concentration *c*_0_ = 1 mol·L^–1^ = 1 M.

### 3.1 Water Monomer (H_2_O)

To verify that we have correctly coupled the NNP library with the engine for performing quantum and classical MD simulations, and to carry out a preliminary evaluation of the accuracy of this combination of methodologies, we first performed simulations of a single neutral water molecule in the gas phase. We take as reference results the reported theoretical IR spectra of Benson et al.^57^ and Plé et al,^38^ both of whom computed the infrared spectrum of a single gas phase water molecule at 300 K using a diversity of approximate and exact methodologies for quantum dynamics and sampling. In particular, they report spectra calculated via: classical MD, thermostated ring polymer MD (TRPMD),^36^ centroid molecular dynamics (CMD),^37^ quasi-centroid molecular dynamics (QCMD),^35^ QTB, various linearized semiclassical initial value representation (LSC-IVR) approaches,^33^ and the discrete variable representation (DVR).^58^ The latter is taken as the exact reference result within the model considered.

In order to affect a direct comparison with the reference results, we do not remove the translational motion of the center-of-mass of the system nor the global rotational motion. As a consequence, we observe that all peaks within the calculated spectra gain an additional subpeak which originates from the combination with global rotations.

The results of the classical and QTB simulations are presented in Figure 1, together with the DVR and TRPMD reference data (extracted from ref 38). The agreement between QTB, TRPMD and the exact reference demonstrates noticeable frequency dependence. The broad band located between 0 cm^–1^ and ∼500 cm^–1^ is reproduced accurately in both position and overall shape by all methods.

**Figure 1:**
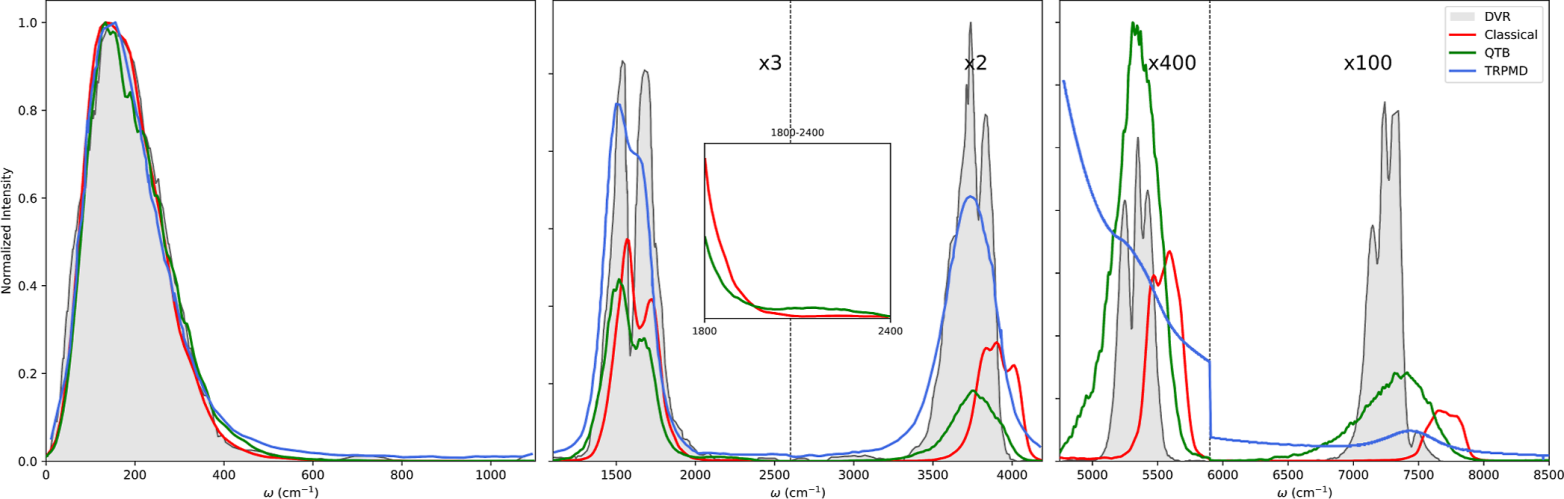
IR spectrum of a single water molecule in the gas phase at 300 K. Classical results are shown in red while the spectrum obtained via QTB is shown in green. The spectrum obtained using the numerically exact discrete variable representation is shown as the filled gray curve, while that obtained via TRPMD is shown in blue. The latter two spectra are adapted from.^38^ All spectra are normalized so that the highest peak of each individual spectrum included in the figure has a maximum value of 1. To recover the absolute intensities of the classical and QTB spectra, scaling factors of 624.53 M^–1^ cm^–1^ and 705.90 M^–1^ cm^–1^ should be applied respectively to the normalized values. The inset shows the weak bend–libration combination band around 2000 cm^–1^ in the QTB spectrum which is absent classically.

On the other hand, considering the frequency range 1200–4500 cm^–1^, we observe that QTB correctly captures the quantum red shift (compared to classical) of the bending mode located at approximately 1600 cm^–1^ and of the stretching feature centered around 3700 cm^–1^. The centers of the bands are in good agreement with the DVR results that, however, present sharper features. Furthermore, the relative intensities of these two peaks are qualitatively preserved, but QTB underestimates their height and fails to resolve the finer features shown by the DVR spectra. This failing is shared by TRPMD (and LSC-IVR results of refs 38 and 57 , not shown here) whose intensities are, however, in somewhat better agreement with the DVR reference. We note that QTB captures the weak bend–libration combination band around 2000 cm^–1^ (see inset of Figure 1). This band is a result of the coupling of the low frequency librational modes with the OH bending mode, located at approximately 1600 cm^–1^. It is known that classical simulations are incapable of reproducing this feature^59^ (see also red curve in the inset). Its presence in the QTB spectrum is a result of the inclusion of NQEs in the calculations together with the accuracy of the dipole moment surface used. TRPMD shows a relatively flat background signal between 2000 and 3000 cm^–1^, hiding the combination band, but it is not clear if this is due to the dynamics or the potential and dipole surfaces.

Turning to the near-infrared region (ω > 4700 cm^–1^), there are two combination bands at ∼5300 cm^–1^ and ∼7300 cm^–1^ which we again observe are correctly downshifted in frequency in the QTB spectra compared to the classical spectra. Benson et al. had previously remarked that the intensities of these peaks are severely underestimated by ring polymer methods, with their intensities no more accurate than the classical results, which they find to drastically undershoot the relative intensities seen in the exact spectrum. Moreover, the authors found that the results obtained via the LSC-IVR method (not shown) gave considerably better agreement of the relative intensities with the exact results. Based on these observations, they concluded that the failure of ring polymer methods in the near-infrared region should not be due to a lack of real-time coherences, as LSC-IVR also neglects these. We find that QTB performs rather differently to TRPMD in this high-frequency region. In particular, it produces appreciable intensity around both the 5300 cm^–1^ and 7300 cm^–1^ bands (in fact, slightly overestimating the former) whereas TRPMD underestimates both and demonstrates considerably greater broadening (a well-known artifact of TRPMD^36^). This observation is aligned with that made in ref 38. In this work, the authors proposed that the explanation for the greater accordance of the (ad-)­QTB results with the exact reference in the near-infrared region is due to the fact that QTB provides a more faithful sampling of the underlying Wigner distribution than ring polymer methods, highlighting the importance of appropriately accounting for correlations in the nuclear phase space distribution as well as coherences in the quantum dynamics.

We note that, although still underestimated, our classical predictions of the relative intensities are noticeably closer to the exact results in this region than those reported in refs 38 and 57 . A possible explanation for this disparity is the different dipole moment surfaces and/or potential energy surfaces used in the different studies.

Even though we are using a different potential and dipole surface, the results described in this section agree with those reported for QTB by Plé et al.^38^ Overall, the water monomer benchmark shows that QTB is able to capture the main qualitative influence of nuclear quantum effects in the spectrum. The low frequency band is reproduced well, the O–H stretching and overtone features are correctly red-shifted relative to the classical spectrum, and the near-IR intensities are more clearly captured than in TRPMD. That being said, the comparison with the DVR reference has notable limitations. In particular, QTB induces considerable broadening in the mid- and near-infrared regions, under-resolving the sharp DVR structure. This unphysical broadening, present also in the TRPMD results, will be discussed more in detail in Section 4.

### 3.2 Protonated Water Dimer/Zundel Cation (H­(H_2_O)_2_^+^)

As the next step-up in complexity, simulations of the gas-phase protonated water dimer H­(H_2_O)_2_^+^ were performed at a temperature of 300 K. To assess the accuracy of our simulations we start by comparing with RPMD calculations performed by Huang et al. at the same temperature.^60^ At variance with the simulations of the monomer, we have removed the global rotation and translational motion of the center-of-mass (COM) of the system in order to match as closely as possible the conditions of this reference work. The results are shown in the left panel of Figure 2. The qualitative agreement between QTB and RPMD is similar to that observed for the monomer. In spite of differences in the intensities of the peaks—in particular at low frequencies where QTB also manifests a more pronounced signal in the range 500–750 cm^–1^—the red shift of the higher frequency peak with respect to classical (and harmonic) results is captured in a very similar fashion by both methods. Note that the classical result shows two closely spaced peaks between ∼3600 cm^–1^ and 3800 cm^–1^—corresponding to OH stretches^32^—that are difficult to discern in the RPMD and absent from the QTB signals. This is again a manifestation of the pathological peak broadening associated with these methods. The right panel of Figure 2 shows QTB and classical spectra for frequencies greater than 4500 cm^–1^. RPMD results are not available in this frequency range. Both our classical MD and QTB results correctly predict the presence of the overtone peaks at around 7500 cm^–1^ (7200 cm^–1^) and 5500 cm^–1^ (5300 cm^–1^) which have been previously reported in an experimental work by McDonald et al.,^61^ and whose locations can also be predicted on theoretical grounds based on the positions of lower-lying fundamentals. The first of these overtones is attributed to twice the frequency of the O–H stretch mode (∼3600 cm^–1^), while the second is attributed to the combination of the O–H stretch and water bending modes (∼1600 cm^–1^–2000 cm^–1^). The combination of the O–H stretch with the asymmetric/shared-proton stretch (1000–1400 cm^–1^) would be expected at approximately the sum of the corresponding fundamentals, i.e. around 4600–5000 cm^–1^ for the present spectra. In the 300 K spectra this region contains only weak, broad intensity and no clearly resolved separate peak. This feature is likely obscured by the low-frequency tail of the stronger O–H stretch/water–bend combination band, which is expected around 5200–5500 cm^–1^. The experimental intensities in this high-frequency region should also be interpreted with some caution. Hammer et al. showed that rare-gas tagging perturbs the Zundel spectrum: Ne tagging preserves the symmetric H_5_O_2_^+^ core more closely, whereas Ar tagging produces stronger solvent-induced symmetry breaking and shifts of both the free O–H stretch and shared-proton bands.^62^

**Figure 2:**
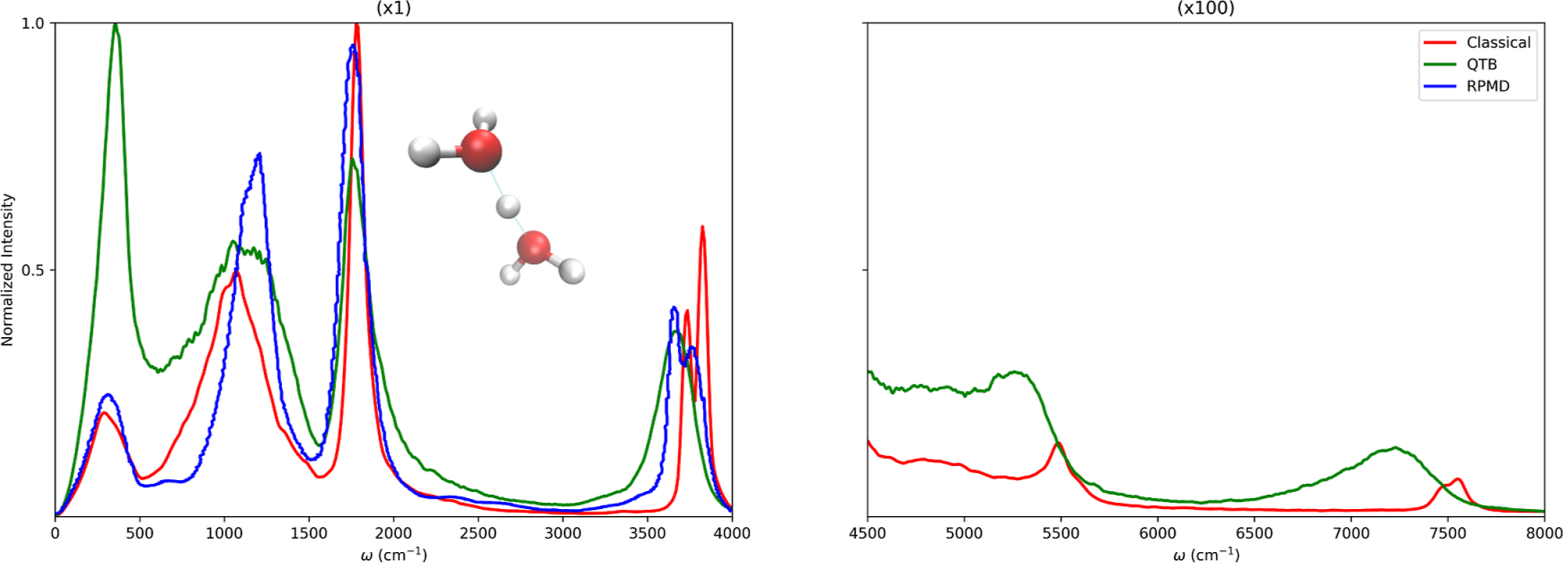
IR spectrum of the protonated water dimer in the gas phase at 300 K. The spectra are normalized so that the highest peak of each individual spectrum included in the figure has a maximum value of 1. To recover the absolute intensities of the classical and QTB spectra, scaling factors of 13.78 M^–1^ cm^–1^ and 6.59 M^–1^ cm^–1^ should be applied respectively to the normalized values. Classical results are shown in red, while the spectrum obtained via QTB is shown in green. The RPMD spectrum in the range 0–4000 cm^–1^ is shown in blue and is adapted from.^60^ We include the RPMD reference result in this figure, since it was also obtained at the higher temperature of 300 K. RPMD is only shown in the left panel since Huang et al. limited their study to frequency range 0–4000 cm^–1^.

We moreover observe that the relative intensities of the peaks show slightly different behavior in our QTB results compared to the RPMD results reported by Huang et al. With QTB, we find that the lowest lying peak, slightly below 500 cm^–1^, (corresponding to wagging, rocking and twisting modes) is, in fact, the most intense peak in the spectrum as opposed to the RPMD and classical spectra where the peak at 1700 cm^–1^ is that with the greatest intensity. Otherwise, the demonstrated trends of the relative intensities are qualitatively identical.

To further evaluate the performance of QTB for this nontrivial system, in Figure 3 we compare results calculated at 20 K with the (zero-temperature) results presented in ref 32 by Yu et al. In this work, the authors performed a series of simulations of the Zundel cation (together with larger clusters) using VSCF/VCI. Results of these calculations were compared with experimental results and reference MCTDH and time-dependent tree tensor network states (td-TTNS) calculations. In particular, the authors carried out VSCF/VCI calculations using an increasing number of modes. This allowed them to demonstrate the importance of including the low frequency modes (wagging, rocking and twisting modes) in order to correctly capture the spectral features of the Zundel cation which previous applications of VSCF/VCI had failed to do. (T)­RPMD results are not available at this lower temperature, most likely due to the large number of replicas required for convergence.

**Figure 3:**
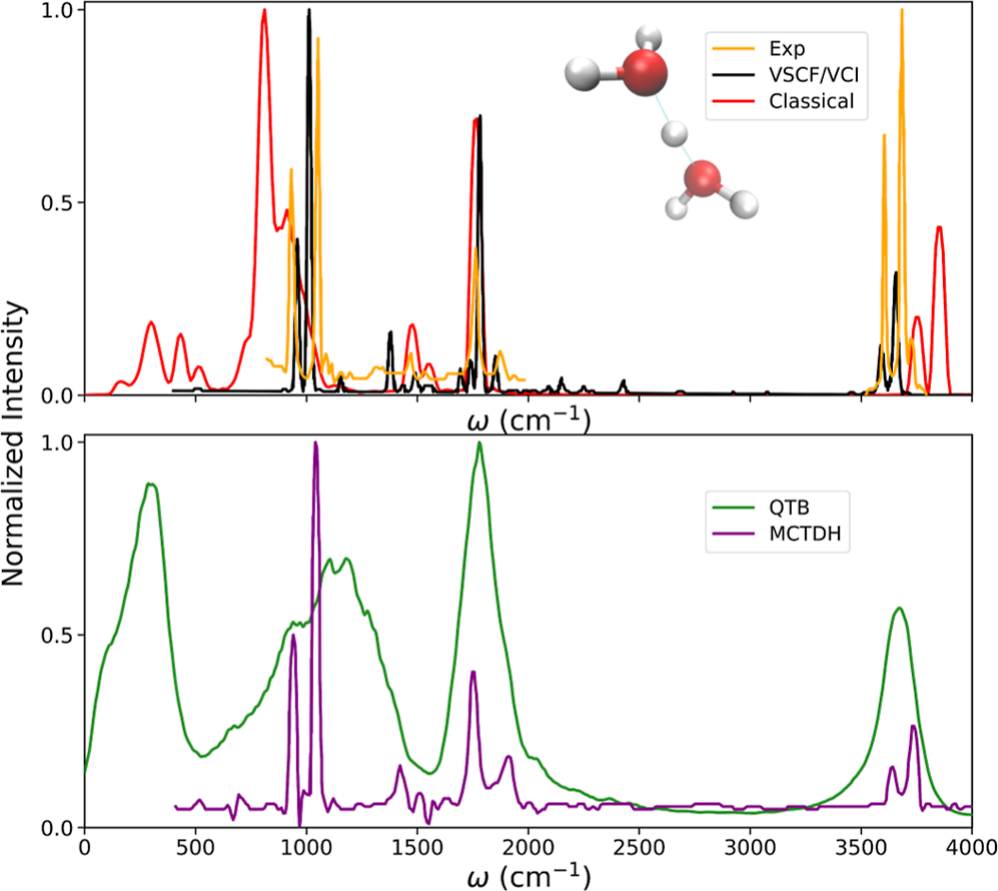
IR spectrum of the protonated water dimer in the gas phase at 20 K. This lower temperature was considered in order to investigate whether some of the finer-grained features observed in the reference high-level theoretical results would emerge in the classical and QTB spectra when thermal effects are reduced. Classical results are shown in red, while the spectrum obtained via QTB is shown in green. Reference spectra obtained from MCTDH calculations (purple curve), VSCF/VCI calculations (black curve), and experiment (orange curve) are adapted from.^32^ The spectra are normalized so that the highest peak of each individual spectrum included in the figure has a maximum value of 1. To recover the absolute intensities of the classical and QTB spectra, scaling factors of 1.99 M^–1^ cm^–1^ and 0.44 M^–1^ cm^–1^ should be applied respectively to the normalized values.

The first observation that can be made concerns the doublet structure observed in previous experimental and high-level theoretical works around 1000 cm^–1^, which, as remarked by Yu et al., has previously been associated with an asymmetric proton stretch mode. Our classical MD result for this mode is red-shifted with a peak around 900 cm^–1^, and the QTB spectra shows a blue-shift (similar to the QTB and RPMD result at 300 K), yielding a peak at around 1200 cm^–1^. In this case too, the QTB spectrum lacks the well resolved doublet-like structure observed in experiments and in the MCTDH spectra at around 900–1000 cm^–1^. This feature is only traceable in the left shoulder of the broad peak centered at these frequencies. The blue shift of the proton-transfer peak can be explained by the nearly quartic form of the potential energy along the asymmetric proton stretch.^63^ The classical dynamics probes only the bottom of the well where the harmonic frequency is low, while the quantum dynamics also probes the repulsive walls. As for the comparison with MCTDH results, it is interesting to note that averaging the MCTDH peaks’ positions with the spectral weights reported in ref 23, we find that the resulting weighted average peak position is in good agreement with that which we observe in our QTB spectrum. Peak broadening in QTB leads to a coalescing of the distinct peaks observed in the MCTDH spectrum.

The broad QTB feature associated with the proton transfer motion extends up to about 1500 cm^–1^, covering the experimental and MCTDH structures around 1400 cm^–1^. The central position of the experimental and MCTDH peaks at 1900 cm^–1^ is well captured, but again with loss of finer features. The broadening of the peaks in the QTB spectra then persists even to these low temperatures, and is a consequence of the zero-point motion, captured by QTB, and which endows the system with a significant “effective temperature” even at 20 K.

To summarize, QTB is able to accurately predict the main spectral features of the Zundel cation. Locations of band centers are in good accordance with reference quantum results obtained at low temperature using MCTDH and VSCF/VCI, while peaks in the spectrum align well with RPMD results computed at 300 K (within the frequency range where reference RPMD data is available). On the other hand, their relative intensities demonstrate some notable differences. QTB fails to capture some of the fine structure predicted by high-level theoretical techniques, such as the shared-proton/proton-transfer doublet near 900–1000 cm^–1^ and the finer features in the O–H stretching region (3500–4000 cm^–1^)—a deficiency which is also observed in the RPMD results. This is ostensibly a consequence of the broadening effect of the thermostat causing these subpeaks to coalesce. Otherwise, we find that even the higher frequency spectral features found in the overtone region of the QTB spectrum are in good agreement with their predicted locations despite the QTB signal exhibiting a rather diffuse baseline.

### 3.3 Protonated Water Trimer (H­(H_2_O)_3_^+^)

For this cluster, see Figure 4, we report results for the temperature of 100 K in order to compare with the thorough spectroscopic analysis reported by Yu and Bowman.^21^ In this study, the authors performed a series of TRPMD, classical MD, double harmonic, and quantum VSCF/VCI^64^ calculations with the goal of comparing the observed spectral features of H­(H_2_O)_3_^+^ with previous experimental results.

**Figure 4:**
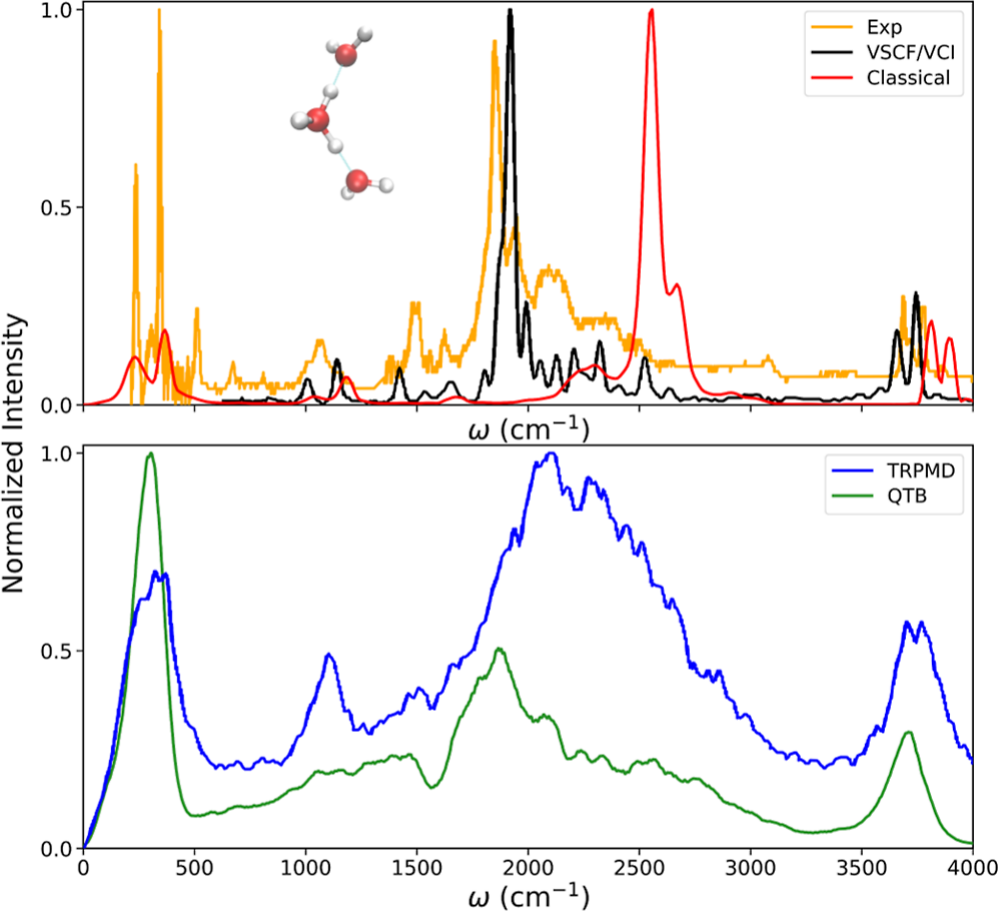
IR spectrum of the protonated water trimer in the gas phase. All simulations were performed at 100 K. Classical results are shown in red while the spectrum obtained via QTB is shown in green. Reference spectra using VSCF/VCI (black), TRPMD with the PILE integrator (blue) and the experimental spectrum (orange) are adapted from.^21^ Each individual spectrum is normalized so that its maximum value adopts a value of unity. To recover the absolute intensities of the classical and QTB spectra, scaling factors of 14186.43 M^–1^ cm^–1^ and 3909.80 M^–1^ cm^–1^ should be applied respectively to the normalized values.

Note that, despite considering a lower temperature of 20 K via classical MD, the reference work only reports TRPMD for the higher temperature of 100 K. This is again due to the considerable computational cost associated with the necessity of employing a larger number of nuclear replicas in the TRPMD simulations at lower temperatures to reach convergence. Similarly to the previous system, we were able to perform QTB simulations of the trimer at the reduced temperature of 20 K without issue. However, given the negligible differences in the resulting spectrum compared to that obtained at 100 K, we opt to show only the latter. In order to achieve satisfactory control of the temperature and zero-point energy leakage, a friction value of γ = 20 ps^–1^ was used in the QTB simulations while in the classical case a value of γ = 2.0 ps^–1^ was found to be sufficient.

The experimental spectrum, orange curve in the upper panel of Figure 4, shows two sharply resolved peaks at low frequencies, followed by a series of features culminating in a broad multipeak feature whose most intense peak is located at 1900 cm^–1^, and finally a high-frequency doublet-like structure in the region 3500–4000 cm^–1^. The central band is known to result from strongly anharmonic excess proton stretching in the hydrated hydronium core mixed with lower frequency motions such as hydronium rotation and umbrella-type distortions.^32,65^ In contrast, the high-frequency features between 3500 and 4000 cm^–1^ are associated with terminal or weakly bonded O–H stretches. VSCF/VCI (black curve in the same plot) results capture quite well the signal for frequencies greater than 500 cm^–1^ with a slight blue shift of the position of the main peak in the central feature. Notably, classical calculations (red curve) are significantly blue-shifted and sharper than experiments. This problem is corrected by the TRPMD results of Yu and Bowman.^21^ and our QTB simulations (blue and green curves in the bottom panel of the figure, respectively). In both cases, the fine features of the band are lost due to broadening, but the position of the main peak is moved closer to the experimental value, with our calculation being slightly more accurate than TRPMD. This is a notable success of combining QTB with this highly accurate NN-PES and NN-DMS. Given that the classical MD results we obtain are in good accordance with those of Yu and Bowman this would suggest that it is the use of QTB which is more likely to be responsible for this superior agreement than it is the particulars of the PES and DMS. Lastly, we note that the position of the high (3500–4000 cm^–1^) and low (0–500 cm^–1^) features in TRPMD and QTB calculations is essentially identical and correctly red-shifted compared to the classical result at high frequencies. For this system, QTB provides a better representation of the relative intensities of the different peaks across the spectrum. As usual, both approaches fail to resolve finer features such as the low and high frequency doublets or the band structure of the central feature (with TRPMD seemingly carrying some more indication of the structures) due to broadening.

To summarize, in agreement with the observations for the monomer and the protonated dimer, we find that QTB reproduces well the major spectral features of the protonated water trimer spectrum. The peak locations predicted by QTB for the most prominent bands in this cluster’s IR spectrum are in generally good agreement with the experimental reference and prior theoretical results using highly sophisticated and computationally expensive methodologies. For one of the principal bands, namely the broad excess proton stretching feature in the ∼1800 cm^–1^–2400 cm^–1^ region, arising from proton stretching modes, we even find that QTB apparently provides a better estimate of the red-shift of this band relative to the classical reference than has previously been found with TRPMD. Similarly to observations made for the dimer system, we once more find that many of the less intense and more fine-grained features which are present in reference VSCF/VCI results are missing in the QTB spectrum due to thermal broadening and amalgamation of these bands in the latter.

### 3.4 Protonated Water Tetramer (H­(H_2_O)_4_^+^) in the Eigen Conformation

To further test the capabilities of our calculations, we simulated the gas phase protonated water tetramer in the Eigen conformation at two different temperatures: 20 K and 100 K. The lower temperature simulations were carried out in order to enable comparison with reference experimental and theoretical works. The theoretical results are again taken from a series of papers by Yu and Bowman^8,21^ which employed TRPMD, classical MD, double harmonic, and VSCF/VCI methods to compute the IR spectra of the protonated Eigen isomer of H­(H_2_O)_4_^+^, while the experimental results obtained at approximately 20 K, and which are also used by Yu and Bowman as a reference, were taken from the works of Esser et al.^66^ and Wolke et al.^67^ As in the case of the protonated water trimer, the reference TRPMD results were limited to the higher temperature of 100 K due to the issue of high computational expense incurred by TRPMD at lower temperatures. As we are primarily interested in the comparison of the QTB results with the reference TRPMD results, we present only the results of our QTB and classical MD simulations at 100 K. The calculated spectra are shown in Figure 5. As in the case of the water trimer, we find that the QTB spectra at 20 K are not appreciably different from those at 100 K, and so we do not present them here.

**Figure 5:**
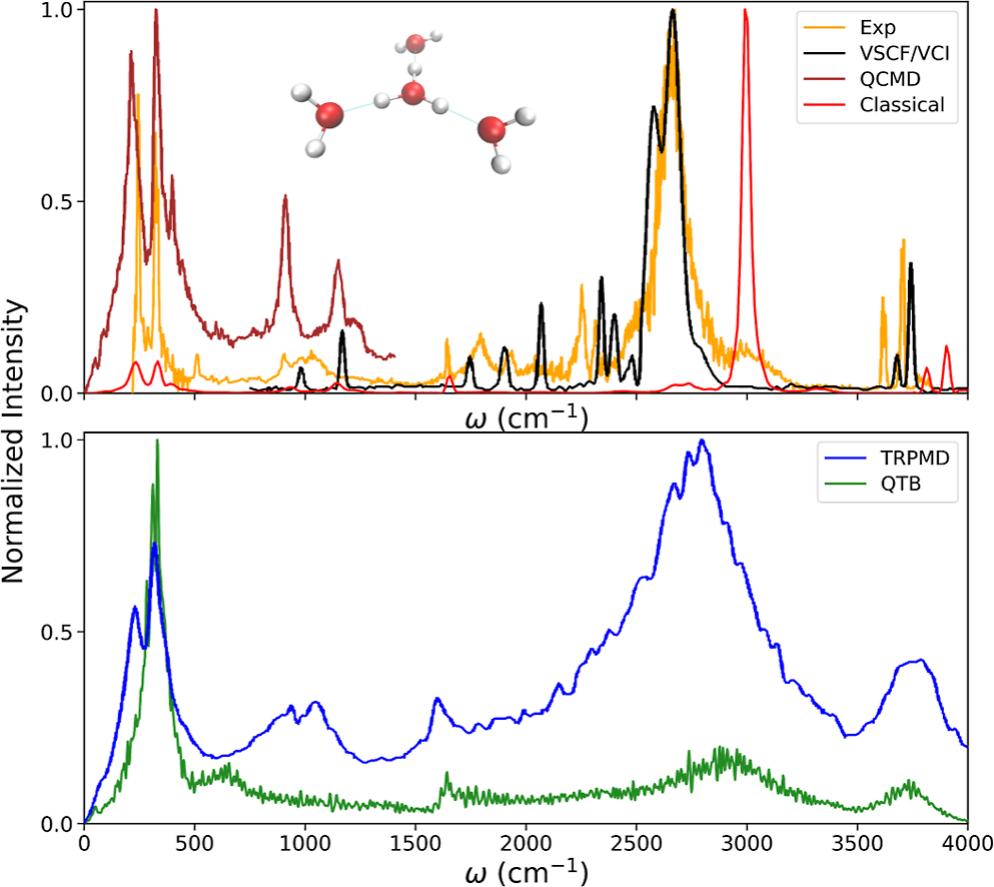
IR spectrum of the protonated water tetramer in the gas phase at 100 K. Classical results are shown in red while the spectrum obtained via QTB is shown in green. Reference spectra using VSCF/VCI (black), TRPMD with the PILE integrator (blue), QCMD (brown), and the experimental spectrum (orange) are adapted from.^21^ Each individual spectrum is normalized so that its maximum value adopts a value of unity. To recover the absolute intensities of the classical and QTB spectra, scaling factors of 32941.24 M^–1^ cm^–1^ and 7141.81 M^–1^ cm^–1^ should be applied respectively to the normalized values.

The spectrum of the protonated water tetramer is very reminiscent of that of the protonated water trimer discussed in the previous subsection. The most prominent feature in the IR spectrum of the tetramer is the peak at around 2650 cm^–1^ (in the experimental spectrum), which has been attributed to the strongly hydrogen-bonded hydronium-core O–H/proton-stretching motion.^21,32^ In the classical spectrum, the corresponding band is located at substantially higher frequency. Similar to the TRPMD results reported by Yu and Bowman,^21^ the QTB results demonstrate a red-shift of this peak with respect to the classical MD and double harmonic results. However, this shift is not sufficiently large to match the experimental location of the peak at around 2650 cm^–1^. Rather, QTB predicts a peak at approximately 2880 cm^–1^, a non-negligible overestimation of this mode’s frequency.

On the other hand, we note that the band in the QTB spectrum corresponding to water stretches at around 3750 cm^–1^ is qualitatively consistent with the TRPMD results of Yu and Bowman, exhibiting a red-shift with respect to the classical MD and double harmonic results by approximately 200 cm^–1^, despite showing rather different relative intensities in the two methodologies. At variance with the experimental result, this band is not split into two distinct peaks in the QTB result, and is instead broadened considerably. This is similar to what the authors of the cited work observe in the case of TRPMD. Similarly, all the lower intensity peaks lying between 1500 and 2500 cm^–1^, which are present in the experimental spectrum, and which are picked up by VSCF/VCI, are not discernible in the QTB spectrum due to broadening and merging of these bands, again like the behavior observed in the case of TRPMD. Likewise, the far-infrared region of the spectrum is not well-resolved by QTB, with only a single broad peak being observed between 0 and 500 cm^–1^. This is in contrast to the reference experimental and QCMD results (see the top panel of Figure 5 and also Figure 7e,g,h in ref 21), which exhibit a number of distinct peaks in this region. These peaks have been attributed to the torsional and stretching motion of the lateral water molecules present in H­(H_2_O)_4_^+^.^66^ The high-frequency QTB signal is nevertheless broad and diffuse, so the comparison should again be regarded as qualitative.

To summarize our analysis of the Eigen cation, QTB captures the qualitative nuclear-quantum red shifts of the main vibrational bands relative to classical MD and predicts the location of principal band centers indicated in the experimental and high-level theoretical spectra with a degree of accuracy comparable to the considerably more expensive TRPMD. However, the agreement with experiment and with high-level reference calculations is limited by substantial broadening and loss of fine structure. As in the previous case of the trimer, the QTB spectrum lacks many of the finer, low-intensity features seen in VSCF/VCI reference results, with these features instead seeming to contribute to broad bands in the frequency ranges in which they are observed in the reference spectra. Regardless, comparison with the classical reference once again indicates the success of QTB in reproducing the characteristic red-shifts of the main vibrational modes when accounting for nuclear quantum effects, with these shifts also clearly visible in the overtone region of the spectrum.

## 4 Conclusions

In this work, we have combined cost-effective QTB dynamics with accurate and efficient machine-learned force fields and dipole moment surfaces to compute the infrared spectra of protonated water clusters. The spectra of clusters of increasing size and complexity were obtained and compared with available experimental data and reference calculations based on classical molecular dynamics, DVR, MCTDH, VSCF/VCI, QCMD and, in particular, (T)­RPMD. Comparisons with path integral based methods are the most relevant for our analysis as these are the most commonly employed finite-temperature approaches within the field. Our results show that QTB faithfully captures typical red-shifts of several peaks in the spectra relative to the classical predictions. Incorporation of NQEs with QTB also allows one to obtain a good description of various combination bands and overtones, predicting band centers to a good degree of accuracy and providing qualitatively reasonable intensities. Although more naturally incorporating thermal effects (especially their influence on dynamics) compared to high-level quantum methodologies like VSCF/VCI and MCTDH, the main limitation of QTB is that it misses some of the fine structure of the spectra due to broadening of the computed signal. The broadening is a well-known pathology in approximate quantum calculations that affects both QTB and, for example, RPMD (or TRPMD). Our results illustrate this very clearly via comparison with the data provided by MCTDH and VSCF/VCI reference calculations. Spectra computed with these methods show considerably sharper features compared to QTB or (T)­RPMD results. MCTDH and VSCF/VCI compute spectra at zero temperature so some thermal or quantum broadening is expected to arise at finite temperatures. However, this effect alone cannot account for the broad line widths observed in the QTB and (T)­RPMD spectra. Different effects contribute—sometimes simultaneously—to the nonphysical broadening reported in this and previous works. First, quantum thermostats employ a—generalized—Langevin evolution that intrinsically broadens peaks. More generally, MD methods that use stochastic or generalized Langevin thermostats to incorporate thermal effects also introduce additional damping and noise into the real-time dynamics, and this thermostated motion can artificially broaden vibrational spectra, a trade-off that is evident in our reported results. The effects of this thermostated motion can be mitigated by deconvoluting the computed spectra as suggested by Ceriotti and co-workers,^55^ but they cannot be completely eliminated, in particular at low frequencies. Second, the broadening—and in particular the diffuse baseline observed in Figure 4 between 700 and 3200 cm^–1^ can arise when the exact spectrum (taken, for example, as the experimental result in the figure) presents several neighboring peaks or multiplet structures. QTB—and also (T-)­RPMD methods—fail to resolve these features and merge them into the broad peak. Similar considerations apply to the tetramer, as demonstrated by the different data reported in Figure 5. Finally, failures of the underlying hypothesis of the QTB or RPMD approximations, e.g. the presence of strong nonlinearity and mixing of modes or considering very low temperatures, can also lead to artifacts. We suspect that this is the origin of the mismatch between our results and those of reference calculations for the water dimer in the low frequency region.

In general, it is worth recalling that both QTB and path integral based calculations of spectra are expected to be more accurate at higher temperatures (closer to the classical limit) or for systems with relatively weak anharmonicity. These limitations notwithstanding, the results reported here confirm that QTB provides useful spectra of essentially the same quality as path integral based results. Crucially, the computational cost of QTB calculations is essentially identical to that of a classical simulation and considerably less than the computational cost of ring polymer MD and alternative methods. The results presented in this paper demonstrate the potential of combining QTB with a high accuracy machine-learned force field and dipole moment surface, and pave the way for future calculations with similar set-ups.

## Figures and Tables

**Figure 6:**
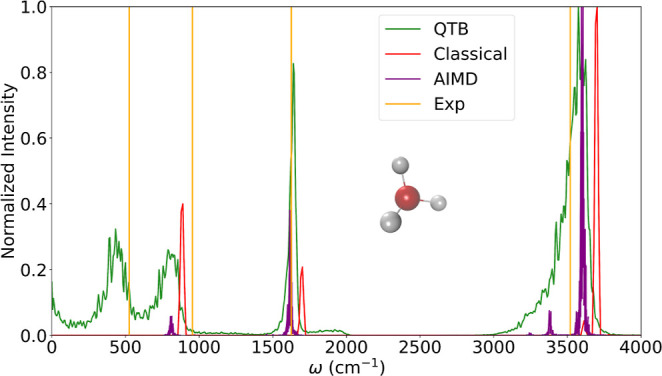
Infrared spectrum of the hydronium ion. Our classical results are shown in red while the spectrum obtained via QTB is shown in green. Reference spectra obtained from AIMD (purple curve) and experiment (orange sticks) are adapted from ref 69 (Figure S3 in the Supporting Information). The spectra are normalized so that the highest peak of each individual spectrum included in the figure has a maximum value of 1. To recover the absolute intensities of the classical and QTB spectra, scaling factors of 0.04 M^–1^ cm^–1^ and 0.26 M^–1^ cm^–1^ should be applied respectively to the normalized values.

**Figure 7:**
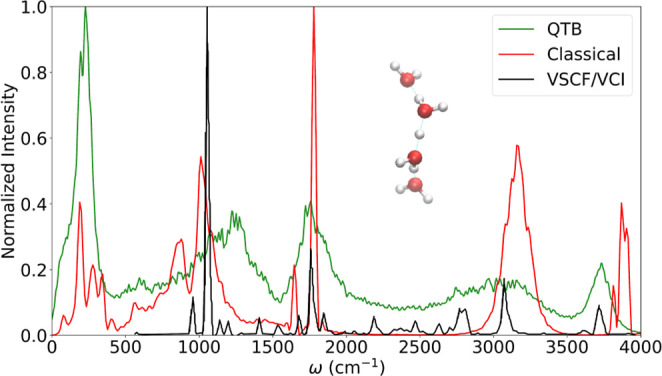
Infrared spectrum of the *cis*-Zundel conformation of H­(H_2_O)_4_^+^ at a temperature of 20 K. Classical results are shown in red while the spectrum obtained via QTB is shown in green. Reference spectra obtained using VSCF/VCI (black curve) is adapted from.^8^ The spectra are normalized so that the highest peak of each individual spectrum included in the figure has a maximum value of 1. To recover the absolute intensities of the classical and QTB spectra, scaling factors of 13.54 M^–1^ cm^–1^ and 7.86 M^–1^ cm^–1^ should be applied respectively to the normalized values.

**Figure 8:**
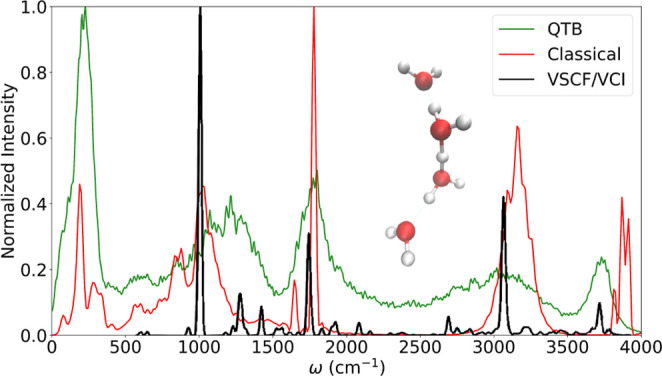
Infrared spectrum of the *trans*-Zundel conformation of H­(H_2_O)_4_^+^ at a temperature of 20 K. Classical results are shown in red while the spectrum obtained via QTB is shown in green. Reference spectra obtained using VSCF/VCI (black curve) is adapted from.^8^ The spectra are normalized so that the highest peak of each individual spectrum included in the figure has a maximum value of 1. To recover the absolute intensities of the classical and QTB spectra, scaling factors of 19.82 M^–1^ cm^–1^ and 9.65 M^–1^ cm^–1^ should be applied respectively to the normalized values.

**Figure 9:**
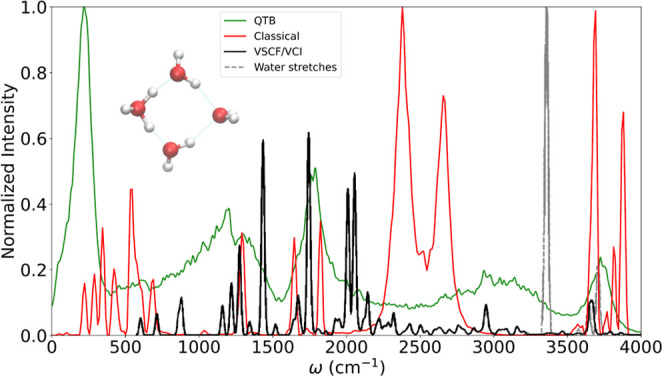
Infrared spectrum of the ring conformation of H­(H_2_O)_4_^+^ at a temperature of 20 K. Classical results are shown in red while the spectrum obtained via QTB is shown in green. Reference spectra obtained using VSCF/VCI (black curve and gray dashed curve showing separately computed water stretches) is adapted from.^8^ The spectra are normalized so that the highest peak of each individual spectrum included in the figure has a maximum value of 1. To recover the absolute intensities of the classical and QTB spectra, scaling factors of 4.70 M^–1^ cm^–1^ and 3.50 M^–1^ cm^–1^ should be applied respectively to the normalized values.

## Appendix A

### Implementing QTB Dynamics

eqs 1–3 define a generalized Langevin dynamics framework, effectively modeling the interaction with an Ohmic bath—i.e., a bath characterized by a spectral density that scales linearly with frequency. In practical implementations, the stochastic force must be prepared in advance of integrating the equation of motion eq 1. The noise is typically generated on-the-fly (OTF), following the method introduced in ref 68 In summary, a white noise signal is first produced for a window of *N*_seg_ timesteps by sampling from a normal distribution with zero mean and unit variance. This signal is then convolved with a kernel derived from the square root of the power spectral density defined in eq 3, yielding a colored noise with the correct frequency characteristics (see ref 49 for detailed methodology). Once computed, the random force is applied throughout the segment before a new noise sample is generated for the next interval. The forces are incorporated into the dynamics using a standard thermostatting approach. In our work, we employed the BAOAB integrator^54^ to implement QTB, owing to its robust theoretical foundation, ease of implementation, and demonstrated compatibility with quantum thermal fluctuations.

As mentioned in the main text, ZPEL was monitored via the deviation from zero of eq 4. No appreciable violation of this condition and/or change in the observables was observed in auxiliary ad-QTB calculations. Those results were therefore not reported.

## Appendix B

### Removing Center-of-Mass Motion in Classical and QTB Simulations of Molecules

If one removes center-of-mass translations and rotations when using QTB, it is important to appropriately adjust the computation of the spectra entering into the quantum fluctuation–dissipation relation. To better understand why this is, consider the following. In a scenario where the translational component of the center-of-mass velocity is removed, the velocities of the individual *N* atoms are modified at each time step according to the following equation

B1
$${ṽ}_{i}\left(t\right)=v_{i}\left(t\right)-\frac{m_{i}}{M}\sum_{j=1}^{N}v_{j}\left(t\right)$$

where $M=\sum_{i=1}^{N}m_{i}$ is the total mass of the system. The two sides of the FDR computed during a QTB simulation are

B2
$$C_{vF,i}\left(\omega \right)$$

B3
$$m_{i}\gamma_{i}\left(\omega \right)C_{vv,i}\left(\omega \right)$$

where *C*_vF,*i*_ is the velocity-random force correlation, and *C*_vv,*i*_ the velocity autocorrelation for atom *i*. If the center-of-mass translational component is removed, then the velocity autocorrelation function *C*_vv,*i*_(ω) is computed using the modified velocities ${ṽ}_{i}\left(t\right)$ . In order to be consistent in the computation of the FDR, it is necessary to also augment the random forces in an analogous fashion. This is achieved via the following modification

B4
$${\mathrm{F̃}}_{i}\left(t\right)=F_{i}\left(t\right)-\frac{m_{i}}{M}\sum_{j=1}^{N}F_{j}\left(t\right)$$

If, instead, one wishes to remove global rotations, then the angular velocities of the individual atoms are modified at each time step according to the following equation

B5
$${ṽ}_{i}\left(t\right)=v_{i}\left(t\right)-\omega_{COM}\left(t\right)\times r_{i}\left(t\right)$$

where **ω**_COM_(*t*) is the angular velocity of the center-of-mass of the system at time *t*. **ω**_COM_ can be computed simply as

B6
$$\omega_{COM}\left(t\right)=I^{-1}\left(t\right)\left[\sum_{i=1}^{N}m_{i}r_{i}\left(t\right)\times v_{i}\left(t\right)-\left(r_{COM}\left(t\right)\times P_{COM}\left(t\right)\right)\right]$$

where **I** ( $I_{kl}=\sum_{i=1}^{N}m_{i}\left(|r_{i}{|}^{2}\delta_{kl}-r_{i,k}r_{i,l}\right)$ with *k*, *l* = *x*, *y*, *z*, the Cartesian components of the atomic positions) is the moment of inertia tensor of the system, **r**_COM_ is the position of the center-of-mass, and **P**_COM_ is the total linear momentum of the system. The term which is subtracted from the sum in the square brackets is the contribution to the angular momentum of the total system arising purely from the translational motion of the center-of-mass. This must be removed in order to isolate the purely rotational part of the total system’s motion.

In the BAOAB scheme, both the B and A step conserve the angular momentum (provided the computed physical forces are accurate enough). The removal of overall angular momentum is thus performed after the update of the velocities in the O step. In order to be consistent in the computation of the FDR, it is necessary to also augment the random forces in an analogous fashion.

This is achieved via the following modification

B7
$${\mathrm{F̃}}_{i}\left(t\right)=F_{i}\left(t\right)-m_{i}\left(r_{i}\left(t\right)\times {\mathrm{\omega ̇}}_{COM}\left(t\right)\right)$$

where ${\mathrm{\omega ̇}}_{COM}\left(t\right)$ is the angular acceleration of the center-of-mass of the system at time *t*. ${\mathrm{\omega ̇}}_{COM}\left(t\right)$ is computed from the torque, τ acting on the system as a result of the random forces

B8
$${\mathrm{\omega ̇}}_{COM}\left(t\right)=I^{-1}\left(t\right)\tau \left(t\right)$$

B9
$$=I^{-1}\left(t\right)\sum_{i=1}^{N}\left[r_{i}\left(t\right)\times F_{i}\left(t\right)-r_{COM}\left(t\right)\times F_{COM}\left(t\right)\right]$$

where again the term which is subtracted from the sum in the square brackets is the contribution to the torque on the system arising purely from the translational motion of the center-of-mass. This must be removed in order to isolate the torque arising purely from the random forces acting on the system.

This modification of the random force is fully consistent with that of the velocities, setting $\omega_{COM}\left(t\right)={\mathrm{\omega ̇}}_{COM}\left(t\right)\times dt$ . In practice, since the QTB random forces are generated in segments of length *N*_seg_, these modifications are performed at the end of each segment, just before computing the correlation functions that enter into the FDR.

## Appendix C

### Infrared Spectra of a Selection of Additional Water Clusters

In this section, we present the infrared spectra of the water clusters H_3_O^+^, H­(H_2_O)_4_^+^ (*trans*-zundel), H­(H_2_O)_4_^+^ (*cis*-zundel), H­(H_2_O)_4_^+^ (ring), which were omitted from the main text for the sake of brevity. The infrared spectra were computed using the same methodology as described in Section 2. All spectra are shown at a temperature of 20 K to facilitate comparison with available zero temperature VSCF/VCI and double harmonic theoretical results and low temperature experimental and AIMD data.

### Hydronium Ion/Protonated Water Monomer (H_3_O^+^)

The infrared spectrum of the hydronium ion is shown in Figure 6. We compare this with the work of Kulig and Agmon.^69^ who report both experimental and theoretical infrared spectra of H_3_O^+^. Their (classical) theoretical spectra were computed at *T* = 50 K using AIMD with the BLYP functional, a double-ζ valence polarization (DZVP) basis set and Goedecker-Teter-Hutter pseudopotential.

Looking first at the far-infrared region, we note the two vibration-inversion bands, ν_2_(1^–^ ← 0^+^) and ν_2_(1^+^ ← 0^+^), are clearly visible in the QTB results in Figure 6 at around 820 cm^–1^ and 450 cm^–1^ respectively. The experimental results reported by Kulig et al. and previous experimental and theoretical results (see ref 70) place the centers of these bands at around 925 cm^–1^ and 525 cm^–1^. On the other hand, the AIMD results of Kulig et al. fail to predict two distinct bands in this region, instead showing a single band at 813 cm^–1^ (see Figure 6). This is likely due to their neglect of nuclear quantum effects. Indeed, given that the ν_2_(1^–^ ← 0^+^) and ν_2_(1^+^ ← 0^+^)) modes arise from umbrella inversion motion of the hydronium ion, it is expected that these modes will be significantly affected by nuclear quantum effects which facilitate tunnelling even at low temperatures. Contrariwise, for these modes to show up in classical simulations, it is necessary to have a sufficiently high temperature to overcome the energy barrier associated with the inversion motion. Indeed, these IR modes do in fact appear in the classical MD spectrum when the temperature is raised to 300 K (not shown).

The next prominent band appearing in the spectrum of the hydronium ion is predicted by experiment to be located at around 1622 cm^–1^. Our QTB results predict a band at ∼1640 cm^–1^ which is in good agreement with this experimental value. Contrariwise, we find our classical results predict the center of this band to be located at closer to ∼1700 cm^–1^ indicating that NQEs act to noticeably red-shift this band. A similar observation is made for the band located at ∼3510 cm^–1^ in the experimental results (corresponding to stretching modes). Our QTB results predict this peak to be located at ∼3580 cm^–1^, although this peak is very broad and extends from ∼3000 cm^–1^ to ∼3800 cm^–1^ which we attribute to the broadening effects of the thermostat. The classical results, on the other hand, predict a main peak in this region at ∼3700 cm^–1^ which again indicates the importance of incorporating NQEs to accurately predict the position of this band.

### H­(H_2_O)_4_^+^ in the *cis*-Zundel and *trans*-Zundel Conformations

IR spectra of the *cis*- and *trans*-Zundel conformations of H­(H_2_O)_4_^+^ are not frequently reported in the literature. This is ostensibly due to the fact that these conformations of H­(H_2_O)_4_^+^ are less energetically favorable than the Eigen conformation discussed in the main text (see Section 3.4). However, the investigation of these conformations (along with the ring conformation discussed next) is important in disentangling the contributions of these conformers to the overall spectrum of H­(H_2_O)_4_^+^. As noted by Kulig et al.^69^ and a subsequent work by Yu and Bowman,^8^ many of the spectral features in the low frequency region of H­(H_2_O)_4_^+^ are not explicable without considering the contributions of these conformers. We therefore present spectra of the *cis*- and *trans*-Zundel conformations of H­(H_2_O)_4_^+^ calculated using QTB and classical MD in Figures 7 and 8 respectively. We include as reference results the VSCF/VCI spectra calculated by Yu and Bowman^8^ (although it should be noted that the VSCF/VCI data was only available in the range 500 cm^–1^ to 4000 cm^–1^).

The spectra of both the *cis*-Zundel and *trans*-Zundel conformations unsurprisingly exhibit many of the same features found in the spectrum of the H_5_O_2_^+^ (Zundel) ion discussed in Section 3.2. Indeed, as noted by Yu and Bowman in ref 8, the dominant band in the spectra of the Zundel isomers is located at 1000 cm^–1^ and is due to stretching motion of the shared proton within the isomers. We find that our classical results also predict a band located at ∼1000 cm^–1^. However, our QTB spectra show this peak to be noticeably blue-shifted to ∼1250 cm^–1^. This is consistent with our previous observations for the Zundel ion (see Section 3.2) where we argued that this blue-shift arose from the sampling of a nearly quartic potential by the shared-proton coordinate.

The water-bending region provides a second useful comparison. The VSCF/VCI spectra of Yu and Bowman show intense bending features in the 1600–1800 cm^–1^ window for the Zundel isomers.^8^ Our classical spectra also contain strong peaks in this range, while the QTB spectra retain intensity in the same window, but with broader line shapes and reduced separation between neighbouring features. In the O–H stretching region, the reference VSCF/VCI spectra contain transitions spread over approximately 3000–3900 cm^–1^. The present spectra likewise show intensity in this high-frequency region, although the QTB bands are broad and do not resolve the individual VSCF/VCI transitions.

### H­(H_2_O)_4_^+^ (Ring Conformation)

We present our calculated IR spectra for the ring conformation of H­(H_2_O)_4_^+^ in Figure 9. As noted by Yu and Bowman, the ring conformation can be thought of as a water molecule hydrogen bonded to an H_7_O_3_^+^ cluster, which results in the ring-like structure. Consequently, one expects to observe many of the features of these two species within the spectrum of the ring isomer. This is indeed the case, and the spectrum of the ring conformation of H­(H_2_O)_4_^+^ exhibits bands at approximately 1450, 1750, 2000, and 3660 cm^–1^ which coincide with those of H_7_O_3_^+^. These bands have been attributed to a water bending mode, hydronium bending mode, hydronium stretching mode, and the OH stretching mode of hydronium, respectively. In addition to these bands, the VSCF/VCI calculations performed by these authors also predict the presence of several less intense features in the frequency ranges bridging the aforementioned bands. The presented QTB spectrum contains intensity in each of these frequency windows, but the correspondence with the VSCF/VCI reference is not one-to-one. In the classical spectrum, several relatively sharp peaks are found in the 1400–2000 cm^–1^ region and in the O–H stretching region above 3500 cm^–1^. In the QTB spectrum, by contrast, the intensity in the 1000–2000 cm^–1^ interval is redistributed into a broad band with maxima near 1200 cm^–1^ and 1750 cm^–1^, while the high-frequency stretching intensity appears as a broad feature spanning roughly 3500-4000 cm^–1^. We therefore avoid assigning the QTB maxima to individual VSCF/VCI transitions. Rather, they should be viewed as finite-temperature, broadened envelopes of several neighbouring anharmonic features. Thus, for the ring conformer, the most direct comparison with the reference calculation is not the position of individual QTB maxima, but the distribution of intensity across the spectral windows assigned by Yu and Bowman: water bending near 1450 cm^–1^, hydronium bending near 1750 cm^–1^, hydronium stretching near 2000 cm^–1^, hydronium O–H stretching near 3660 cm^–1^ and water stretching near 3350 cm^–1^. QTB places intensity in these regions, but merges much of the mid-frequency structure into broad bands.

## Appendix D

### Timings

As an illustration of the computational efficiency of the QTB and adQTB methods, we present in Table 1 the average timings per MD step for each of the 8 different water clusters considered, and each of the three different methods (classical MD, QTB, and adQTB) as well as an estimation of the total MD trajectory length that can be attained on standard HPC hardware in 24 h of wall time. The data for adQTB is provided for completeness, even though—as previously explained—the use of this method was not necessary for the spectra presented in the paper. The simulations were performed on a single core of an Intel­(R) Xeon­(R) Platinum 8360Y CPU @ 2.40 GHz. The timings were averaged over the entire trajectory length of 1 ns, and the number of steps taken was 10^7^. The scaling with system size is approximately linear, with QTB and adQTB generally taking around the same wall time per MD step as classical MD. As a consequence, QTB constitutes a viable method for the inclusion of NQEs in larger molecular systems, facilitating access to the long simulation times necessary to accumulate sufficient statistics for accurate resolution of IR spectra.

**Table 1:** Average Timings per MD Step for Each of the Eight Different Water Clusters Considered, Using Three Different Methods (Classical MD, QTB, and adQTB)^a^

molecule name	method	Avg time per step (s)	ns per day
H_2_O	classical	0.000116	86.207
H_2_O	QTB	0.000115	86.957
H_2_O	adQTB	0.000107	93.458
H_3_O^+^	classical	0.000142	70.423
H_3_O^+^	QTB	0.000146	68.493
H_3_O^+^	adQTB	0.000143	69.930
H(H_2_O)_2_^+^	classical	0.000317	31.549
H(H_2_O)_2_^+^	QTB	0.000322	31.055
H(H_2_O)_2_^+^	adQTB	0.000316	31.646
H(H_2_O)_3_^+^	classical	0.000546	18.315
H(H_2_O)_3_^+^	QTB	0.000568	17.609
H(H_2_O)_3_^+^	adQTB	0.000549	18.221
H(H_2_O)_4_^+^ (Eigen)	classical	0.000962	10.398
H(H_2_O)_4_^+^ (Eigen)	QTB	0.000776	12.890
H(H_2_O)_4_^+^ (Eigen)	adQTB	0.000852	11.737
H(H_2_O)_4_^+^ (*trans*-zundel)	classical	0.000807	12.384
H(H_2_O)_4_^+^ (*trans*-zundel)	QTB	0.000848	11.789
H(H_2_O)_4_^+^ (*trans*-zundel)	adQTB	0.000850	11.765
H(H_2_O)_4_^+^ (*cis*-zundel)	classical	0.002526	3.956
H(H_2_O)_4_^+^ (*cis*-zundel)	QTB	0.002931	3.409
H(H_2_O)_4_^+^ (*cis*-zundel)	adQTB	0.002941	3.400
H(H_2_O)_4_^+^ (ring)	classical	0.000587	17.012
H(H_2_O)_4_^+^ (ring)	QTB	0.000852	11.737
H(H_2_O)_4_^+^ (ring)	adQTB	0.002258	4.428

a The last column indicates the duration (in nanoseconds) of the MD trajectory that can be simulated per day on a single core of an Intel­(R) Xeon­(R) Platinum 8360Y CPU @ 2.40 GHz.
